# Hodgkin lymphoma with hypereosinophilia in a pediatric patient: case report and review of the literature

**DOI:** 10.3389/fped.2026.1688168

**Published:** 2026-05-20

**Authors:** Rosa Margarita Cruz Osorio, Regina M. Navarro-Martin del Campo, Wangky Carolina Carrasco-Rivera, Lisette Paola Bruijnzeels-Ponce, Jesús Alejandro Gutiérrez-Ortiz, Hannali Quintero-Buenrostro, Violeta Salceda-Rivera, Manuel Donovan Martinez-Albarran, Diego Ugalde-Aviña, Veronica Soto Chavez, Fernando Sánchez-Zubieta, Oscar Gonzalez-Ramella

**Affiliations:** 1 Pediatric Hematology and Oncology Department, Hospital Civil of Guadalajara “Dr. Juan I. Menchaca”, Guadalajara, Jalisco, Mexico; 2 Center for Health Sciences, University of Guadalajara, Guadalajara, Jalisco, Mexico; 3 Pathology Department, Hospital Civil of Guadalajara “Dr. Juan I. Menchaca”, Guadalajara, Jalisco, Mexico

**Keywords:** case report, eosinophils, Hodgkin lymphoma, hypereosinophilia, pediatric cancer

## Abstract

**Background:**

Hodgkin lymphoma (HL) comprises 6% of pediatric cancers, showing bimodal incidence in adolescence/young-adult and over 50 years. Characterized by Reed-Sternberg cells, HL is classified as classic/nodular lymphocytic predominant by WHO. Hypereosinophilia (>1,500/µL eosinophils) occurs in 15% of HL cases.

**Case report:**

A 17-year-old female presented with weight loss, night sweats, and malaise. Examination showed enlarged lymph nodes, splenomegaly, and palmoplantar erythema. Bloodwork revealed eosinophilia, hypoalbuminemia, and elevated lactate dehydrogenase levels. Bone marrow confirmed eosinophilic predominance. Lymph node biopsy diagnosed nodular sclerosis classical Hodgkin lymphoma (NS-cHL). PET-CT scan identified cervical, mediastinal, abdominal and spleen tumoral activity, classified as classical Hodgkin lymphoma or nodular lymphocyte-predominant Hodgkin lymphoma according to WHO criteria. OEPA/COPDAC treatment initiated, follow-up PET-CT showed incomplete metabolic response. She subsequently underwent radiotherapy, achieving complete metabolic response after treatment.

**Conclusion:**

This rare case illustrates profound hypereosinophilia concomitant with HL. Despite an extensive literature search, similar presentations are rarely reported in the literature. This case highlights an uncommon association between classical Hodgkin lymphoma and unusually high levels of hypereosinophilia, supported by the clinical course and the marked decline in eosinophil counts after lymphoma-directed therapy.

## Introduction

Classical Hodgkin lymphoma (HL) is a neoplasm that predominantly affects adolescents and young adults, accounting for approximately 10%–15% of all lymphomas (1). Due to advancements in chemotherapy, radiation therapy, and targeted immunotherapy, it is now associated with a favorable prognosis. The NS-cHL is the most prevalent, representing 70% of HL cases (2). While the diagnosis of HL is generally straightforward, atypical clinical presentations—such as profound hypereosinophilia—can pose significant diagnostic challenges.

Mild eosinophilia is observed in approximately 15% of HL cases; however, extreme hypereosinophilia—defined as an eosinophil count exceeding 1.5 × 10⁹/L—is exceedingly rare (3, 4). This condition may arise from various causes, including parasitic infections (e.g., *Strongyloides stercoralis*), allergic or atopic diseases, adverse drug reactions, and paraneoplastic syndromes associated with myeloproliferative or lymphoproliferative disorders (5). In HL, hypereosinophilia may result from cytokines such as IL-3, IL-5, and GM-CSF produced by malignant cells, which promote eosinophil differentiation and survival (6). These cytokines may also influence the tumor microenvironment, suggesting potential diagnostic and therapeutic implications.

Although eosinophilia has been reported in HL, the extreme levels observed in this case are rarely reported, making it a noteworthy clinical finding. This report describes the case of a 17-year-old female with NS-cHL, who presented with profound hypereosinophilia. By highlighting the clinical and pathological complexities of this rare presentation, this case emphasizes the need for heightened awareness of HL in patients with unexplained hypereosinophilia, particularly in pediatric and adolescent populations. Ultimately, this report aims to broaden the clinical perspective on hypereosinophilia as a potential presentation of HL, reinforcing the importance of considering oncologic causes early in the diagnostic process.

## Case report

A 17-year-old Hispanic female was admitted with a six-month history of symptoms, including profuse diaphoresis and unintentional weight loss of more than 10% of her body weight. She also reported pruritus affecting her hands and had developed fixed, non-tender submandibular and bilateral cervical lymphadenopathies, each measuring approximately 9–10 cm in diameter. There was no evidence of dysphagia or airway compression; however, splenomegaly and palmoplantar erythema were noted on physical examination. There was no prior personal or family history of asthma, allergic diseases, atopy, or drug-related hypersensitivity.

Upon admission, laboratory testing revealed a hemoglobin level of 10.49 g/dL, a hematocrit of 34.5%, and a leukocyte count of 104,900/µL. The leukocyte differential showed neutrophils at 2,570/µL, lymphocytes at 3,130/µL, and marked eosinophilia of 97,880/µL, corresponding to 93.3% of the total white blood cell count. The platelet count was 354,400/µL (Table 1). Additional biochemical tests revealed a lactate dehydrogenase (LDH) level of 201 U/L and a serum albumin concentration of 2.90 g/dL. A structured diagnostic evaluation was performed to exclude secondary causes of hypereosinophilia, with negative results for infectious etiologies, including Epstein–Barr virus, VDRL, HIV, herpes simplex virus, rubella, cytomegalovirus, and Toxoplasma gondii. No clinical features suggestive of autoimmune disease were identified.

**Table 1:** Hematologic evolution and resolution of extreme hypereosinophilia during lymphoma-directed therapy.

Time point	Hemoglobin (g/dL)	Leukocytes (/µL)	Eosinophils (/µL, %)	Platelets (/µL)	Clinical interpretation
At diagnosis	10.49	104,900	97,880 (93.3%)	351,400	Severe hypereosinophilia with leukocytosis; baseline disease burden
Start of 1st OEPA	9.97	68,720	62,120 (90.4%)	268,200	Persistent marked hypereosinophilia prior to treatment effect
Start of 2nd OEPA	9.65	5,140	320 (6.2%)	295,900	Rapid decline in eosinophils; early hematologic response
Start of 1st COPDAC	11.2	21,000	10,600 (50.4%)	254,000	Transient eosinophil rebound during treatment transition
Start of 2nd COPDAC	12.9	10,300	49 (0.47%)	191,000	Resolution of hypereosinophilia
Start of 3rd COPDAC	12.7	6,320	70 (1.14%)	193,100	Sustained normalization of eosinophil count
Start of 4th COPDAC	12.3	5,740	100 (1.72%)	226,500	Stable hematologic recovery
Start of radiotherapy	12.41	3,880	160 (4.15%)	278,100	Mild eosinophilia without clinical significance
End of radiotherapy	13.3	3,510	255 (7.26%)	240,000	Complete hematologic recovery; no recurrence of hypereosinophilia

OEPA, prednisone, vincristine, doxorubicin, etoposide; COPDAC, prednisone, dacarbazine, vincristine, cyclophosphamide.

**Figure 1:**
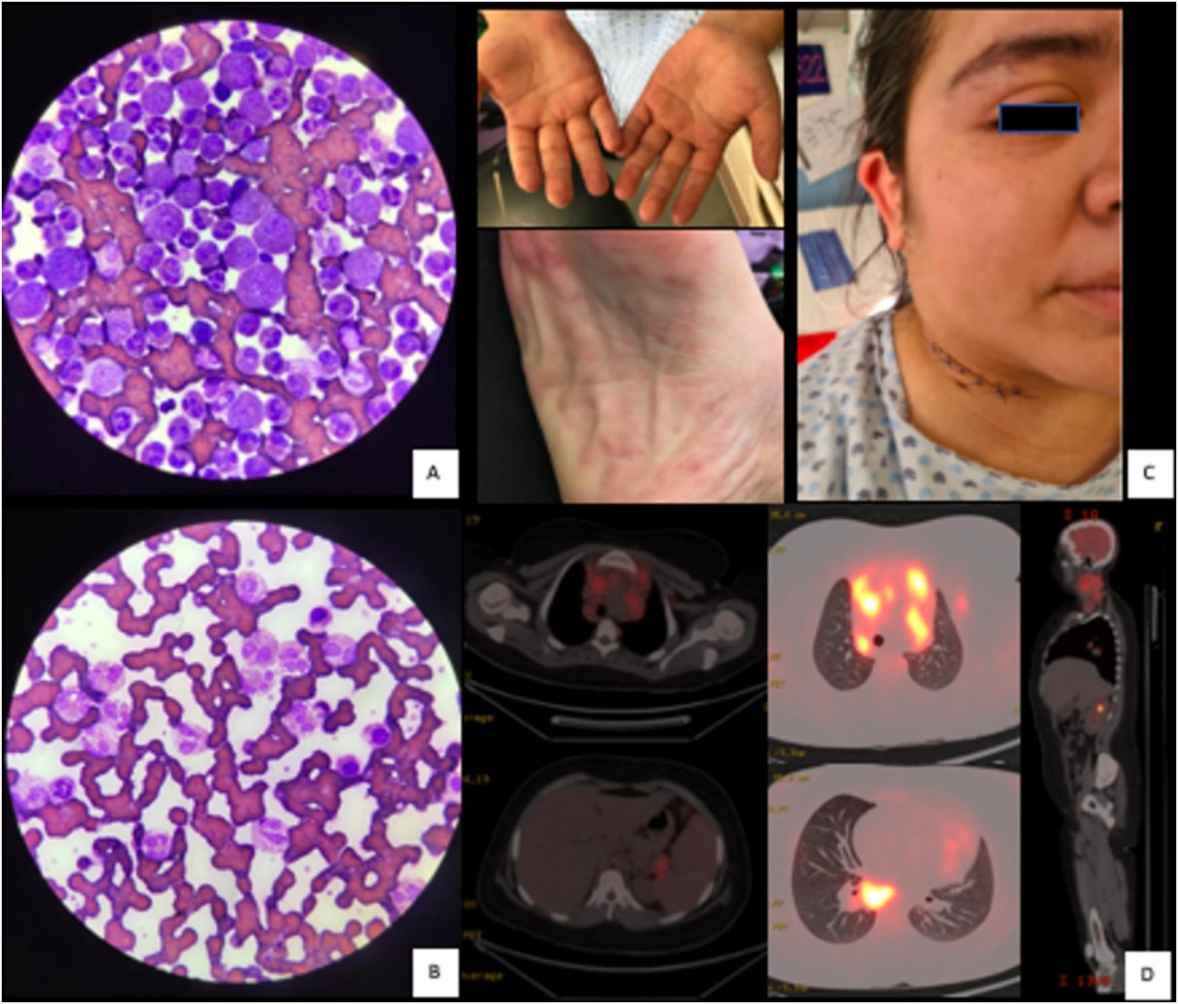
Marked eosinophilia in bone marrow aspirate **(A)** and peripheral blood smear **(B)**, clinical presentation showing extensive cervical lymphadenopathy and palmoplantar erythema **(C)**, and PET-CT scan demonstrating supra- and infradiaphragmatic tumor activity with splenic involvement, consistent with an incomplete metabolic response (Deauville score 4) **(D)**.

A peripheral blood smear confirmed marked eosinophilia (Figure 1B), with other cell lines appearing morphologically normal. A bone marrow aspirate revealed a hypocellular marrow predominantly composed of eosinophilic lineage cells at various stages of maturation—from promyelocytes and myelocytes to metamyelocytes and mature eosinophils (Figure 1A). A bone marrow trephine biopsy was not performed, as PET-CT imaging did not suggest marrow involvement. No blast cells or infiltrative malignancies were identified in the aspirate.

A plain chest x-ray demonstrated a large mediastinal mass accompanied by diffuse pulmonary infiltrates. Computed tomography (CT) revealed conglomerated lymph node clusters with altered morphology across multiple cervical chains, as well as multiple adenopathies in the para-aortic, paratracheal, and aortopulmonary window regions. These findings were associated with splenomegaly and similar lymph node involvement in the retroperitoneum, mesentery, and splenic hilum. A diagnostic positron emission tomography (PET-CT) scan showed abnormal metabolic activity, including bilateral SUVmax of 16.6 at cervical levels II to VI (up to 62 mm), a mediastinal SUVmax of 12 with a metabolic volume of 181 cc at 29%, and splenic lesions with an SUVmax of 5.3 and a maximum diameter of 159 mm (Figure 1D). Abdominal adenopathies at the splenic hilum exhibited an SUVmax of 11.9 and measured 29 mm in diameter. Given the presence of extensive lymphadenopathy and a large mediastinal mass, a lymphoproliferative disorder was strongly suspected, and an excisional lymph node biopsy was prioritized to establish a definitive diagnosis.

**Figure 2:**
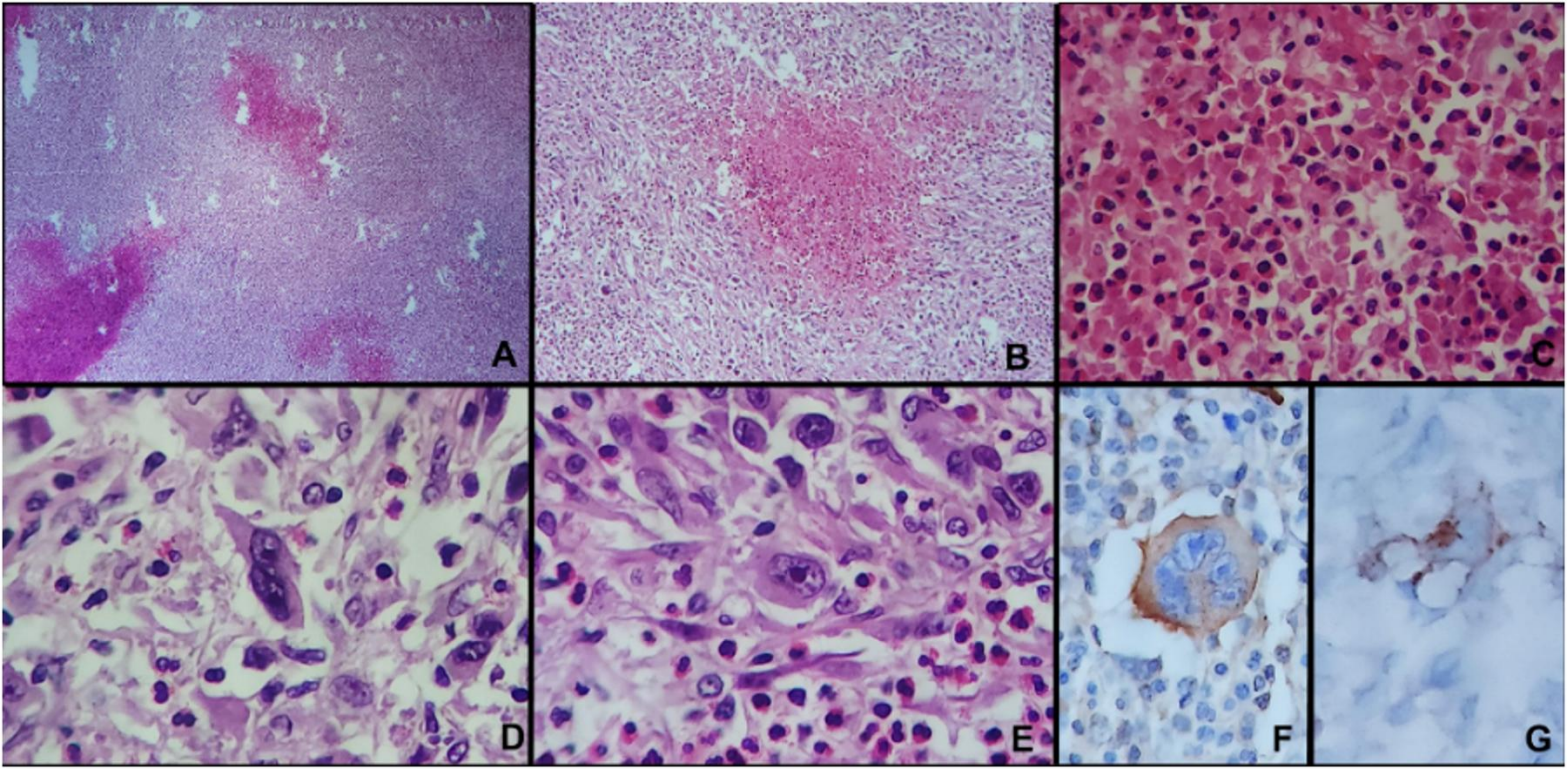
Histopathological and immunohistochemical findings of nodular sclerosis classical hodgkin lymphoma. **(A–C)** Low- and intermediate-power views showing nodular architecture with fibrous bands and a mixed inflammatory background. **(D,E)** High-power views demonstrating characteristic Reed–Sternberg cells within a prominent eosinophil-rich inflammatory infiltrate. **(F,G)** Immunohistochemistry showing positivity of neoplastic cells for CD30 and CD15. Additional immunohistochemical markers, including LMP1 and CD68, were evaluated but are not included in this figure.

A lymph node biopsy confirmed the diagnosis of nodular sclerosis classical Hodgkin lymphoma (NS-cHL) (Figures 2A–G). Immunohistochemical analysis demonstrated that neoplastic cells were positive for CD30 and CD15 (Figures 2F,G) and negative for CD3 and CD20. LMP1 expression was also identified. CD68 staining highlighted the reactive histiocytic component of the tumor microenvironment, with no expression in neoplastic Reed–Sternberg cells. At the tissue level, there was prominent eosinophilic infiltration, with 148 eosinophils per high-power field (HPF; 400  ×   magnification). Genetic testing revealed no mutations in markers such as JAK2 or BCR-ABL.

Based on these findings, the patient was classified as stage IIIBs according to the Ann Arbor staging system, as modified by the Cotswolds criteria. This classification was supported by clinical features including a bulky thoracic mass, splenomegaly, profound weight loss, profuse sweating, hypoalbuminemia, and extreme hypereosinophilia, all of which placed her in the high-risk category.

Treatment was initiated following the OEPA/COPDAC protocol. The patient received two cycles of the OEPA regimen (prednisone, vincristine, doxorubicin, etoposide), followed by an interim PET-CT scan to assess treatment response. She then underwent four cycles of the COPDAC regimen (prednisone, dacarbazine, vincristine, cyclophosphamide).

The interim PET-CT scan demonstrated a reduction in abnormal radiotracer uptake in the neck and mediastinum, along with resolution of splenic lesions and abdominal adenopathies, consistent with an incomplete metabolic response (Deauville score 4) after OEPA induction. Based on these findings, consolidative radiotherapy was indicated according to established OEPA/COPDAC response-adapted treatment criteria, as implemented in our institutional protocol. Hypereosinophilia also markedly decreased following chemotherapy. Following completion of radiotherapy, a post-treatment PET-CT scan demonstrated complete metabolic response, defined as Deauville score 1–2. Although the complete formal imaging report was not fully retrievable, this assessment was confirmed through multidisciplinary clinical evaluation and is supported by the sustained clinical remission and normalization of hematologic parameters.

## Discussion

Eosinophils, a subset of white blood cells, originate in the bone marrow. Cytokines such as IL-3, IL-5, and GM-CSF regulate their maturation and differentiation, with IL-5 playing a particularly prominent role in eosinopoiesis. These cells store and release bioactive compounds that play a key role in infections, allergic reactions, and inflammatory processes (5). In neoplastic diseases, hypereosinophilia may result from cytokine production by malignant cells, as observed in Hodgkin lymphoma (HL), T-cell lymphomas, and acute lymphoblastic leukemia. In our patient, the extreme degree of peripheral eosinophilia was temporally associated with the diagnosis of Hodgkin lymphoma and may reflect a reactive cytokine-driven response mediated by the tumor microenvironment. However, a definitive causal relationship cannot be established from a single case. This interaction suggests a potential role for eosinophils in tumor microenvironment modulation and disease progression. An apparent discrepancy between the hypocellular bone marrow aspirate and the marked peripheral leukocytosis with eosinophilia may be explained by a cytokine-driven process. In Hodgkin lymphoma, cytokines such as IL-5 can promote peripheral eosinophilia without necessarily reflecting marrow hypercellularity (5, 6).

From a clinical perspective, eosinophilia in Hodgkin lymphoma has been most commonly reported in association with the classical subtype, particularly the nodular sclerosis variant, which is characterized by a prominent inflammatory microenvironment and fibrotic remodeling (7, 8). Several studies have suggested that tissue eosinophilia may correlate with adverse features, including bulky mediastinal disease, B symptoms, and increased fibrosis, potentially reflecting a cytokine-driven tumor microenvironment, particularly mediated by interleukin-5 and other eosinophil-stimulating factors (5, 7). Although the prognostic significance of peripheral hypereosinophilia remains controversial, some reports have linked marked eosinophilia with more aggressive disease behavior and suboptimal response to initial therapy (7, 9).

Our patient presented with multiple high-risk features, including bulky thoracic disease, B symptoms, and extreme hypereosinophilia, raising the question of whether the degree of eosinophilia may be associated with treatment response. The incomplete metabolic response observed after OEPA induction (Deauville score 4) could hypothetically be influenced by the underlying tumor microenvironment; however, a causal relationship cannot be established based on a single case. Therefore, hypereosinophilia in this setting should be interpreted as a potential marker of disease biology rather than a direct determinant of treatment resistance. Further studies are required to clarify its prognostic role and clinical implications.

In the context of response-adapted treatment strategies for pediatric Hodgkin lymphoma, the management of patients with incomplete metabolic response after induction therapy remains an area of clinical relevance. Current OEPA/COPDAC-based protocols, including those adopted by cooperative groups in Latin America, aim to reduce long-term treatment-related toxicity by limiting the use of radiotherapy; however, consolidative radiotherapy is recommended in patients with inadequate early metabolic response, typically defined by a Deauville score ≥4 (1, 2). In this setting, our patient met criteria for radiotherapy following OEPA induction, and this approach is consistent with established treatment strategies.

Alternative approaches, including the use of brentuximab vedotin or other salvage regimens, are generally reserved for patients with relapsed or progressive disease rather than those with incomplete early response (1, 2). In our case, the absence of disease progression and the presence of a partial metabolic response supported the continuation of protocol-directed therapy with consolidative radiotherapy rather than escalation to second-line treatment. This decision reflects a balance between treatment efficacy and long-term toxicity, particularly relevant in pediatric populations, where minimizing long-term toxicity remains a central therapeutic goal.

Hypereosinophilia, while relatively common in various pathological processes such as hematologic disorders, infections, allergic diseases, and neoplasms, should always prompt a thorough diagnostic evaluation (5, 10). The diagnostic algorithm proposed in the World Health Organization's 2016 Classification of Eosinophilic Disorders recommends integrating blood and bone marrow morphology, cytogenetics, and immunophenotyping to identify clonal or reactive causes (6). In our patient, the initial workup included peripheral smear review, bone marrow aspirate morphology, and negative JAK2 and BCR-ABL testing. However, additional studies to fully exclude primary or clonal hypereosinophilic syndromes, such as FIP1L1-PDGFRA rearrangements and lymphocytic variants, were not available. Despite this limitation, the absence of morphologic features suggestive of a primary eosinophilic neoplasm, together with the marked decrease in eosinophilia following lymphoma-directed chemotherapy, strongly supports a reactive, cytokine-mediated process in this patient.

In the context of Hodgkin lymphoma staging, PET-CT has largely replaced routine bone marrow biopsy, particularly in the absence of imaging findings suggestive of marrow involvement. This approach is increasingly adopted across multiple centers to reduce unnecessary invasive procedures without compromising diagnostic accuracy. In our patient, the absence of PET-CT evidence of bone marrow disease supported the decision not to perform a trephine biopsy. In this case, assessment of treatment response relied on a combination of interim PET-CT findings, clinical evolution, and hematologic response. Although complete imaging documentation was not fully available, the observed clinical course—including resolution of hypereosinophilia and sustained remission—supports a complete metabolic response following radiotherapy. This integrated approach is consistent with real-world clinical practice, particularly in retrospective case analyses.

Lykkergaard's study of 356,196 individuals established a significant association between unexplained eosinophilia and the subsequent development of Hodgkin lymphoma and myeloproliferative disorders. This finding reinforces the need for prompt evaluation of hypereosinophilia to enable early diagnosis, which is associated with improved prognosis in these malignancies (9). Approximately 10% of hypereosinophilia cases are paraneoplastic in origin, although its prognostic significance remains controversial. Some studies suggest a correlation with poor outcomes, whereas others do not (11). Von Wasielewski, in a study of 1,511 biopsies from patients with HL, found that prominent tissue eosinophilia correlated with poor treatment response in the NS-cHL, although no similar correlation was observed with peripheral eosinophilia (7).

The incidence of eosinophilia in Hodgkin lymphoma is approximately 15%, and it can be detected in both peripheral blood and bone marrow (8). A correlation has been suggested between eosinophilia and NS-cHL, possibly due to eosinophil production of TGF-β and the subsequent stimulation of fibroblasts (9). Moreover, a protein expressed by eosinophilic granulocytes, known as eosinophil cationic protein (ECP), can inhibit proteoglycan degradation in fibroblasts, contributing to the formation of fibrotic bands characteristic of NS-cHL histology (9). In addition, eosinophilia appears to increase the expression of a ligand for CD30, a member of the TNF/nerve growth factor family, which may stimulate Hodgkin Reed–Sternberg cell proliferation (9). These mechanisms highlight the potential role of eosinophils in disease progression and modulation of the tumor microenvironment in HL.

The association between Hodgkin lymphoma and eosinophilia has been recognized for many years. However, its specific role in pathogenesis and its impact on survival remain incompletely understood (12). In our case, CD68 positivity was confined to the reactive histiocytic background and was not identified in neoplastic Reed–Sternberg cells. The presence of focal caseating necrosis raised the possibility of infectious etiologies, particularly tuberculosis; however, acid-fast bacilli staining was negative, and no clinical or laboratory findings supported an infectious process. These findings are therefore best interpreted in the context of the tumor microenvironment rather than an alternative infectious or neoplastic process.

To our knowledge, cases with such extreme levels of hypereosinophilia are rarely reported in the literature, particularly in adolescent patients with NS-cHL. This case is therefore noteworthy for the extraordinarily high eosinophil count, a phenomenon rarely reported in the literature, particularly in adolescent patients with classical Hodgkin lymphoma.

Morena Urbina reported a case of a 19-year-old female with chronic atopic dermatitis, severe pruritus, insomnia, anxiety, lymphadenopathy, leukocytosis (39,700/µL), and eosinophilia (1,580/µL). Lymph node biopsy showed immunostaining positive for BCL2, BCL6, CD3, CD30, and CD15, and negative for PAX5 and CD20, consistent with NS-cHL. Similarly, González Paredes described a 12-year-old female with vomiting, abdominal distension, anuria, lymphadenopathy, and a mesogastric mass. Laboratory tests revealed leukocytosis (32,800/µL), eosinophilia (27,552/µL), and markedly elevated urea (323 mg/dL) and creatinine (23.8 mg/dL). The diagnosis was lymphocyte-predominant NS-cHL, with immunophenotype CD20-, CD30+, CD45+, CD15+, CD57+, and complicated by renal insufficiency at presentation. Sanju Cyriac reported an 8-year-old male with lymphadenopathy, fever, cough, hepatosplenomegaly, superior vena cava obstruction, pleural effusion, leukocytosis (18,000/µL), and eosinophilia (14,220/µL). Bone marrow analysis confirmed eosinophilia, and lymph node biopsy revealed mixed-cellularity HL, which responded to ABVD chemotherapy (8).

Voeller J (13) reported two pediatric cases with hematologic malignancies and severe hypereosinophilia: a 7-year-old female with acute lymphoblastic leukemia complicated by myocardial infarction and stroke, and a 13-year-old female with eosinophilic leukemia, anemia, cardiomyopathy, and pulmonary hypertension (13). Another reported patient had FIP1L1–PDGFRA positivity and was treated with imatinib and corticosteroids. In adults, cases of HL with hypereosinophilia have been associated with tissue damage, particularly cardiac involvement, including hypereosinophilic myocarditis (14, 15). Neuropsychiatric symptoms have also been described in association with marked eosinophilia. A comparison with previously reported cases of Hodgkin lymphoma associated with hypereosinophilia is summarized in Table 2.

**Table 2:** Reported cases of Hodgkin lymphoma associated with hypereosinophilia.

Author (Year)	N	Age/Sex	Histological subtype	Primary site/Disease extent	Stage	Eosinophil count (/µL)	Treatment	Response	Follow-up/Outcome
Urbina et al.	1	19/F	NS-cHL	Peripheral lymphadenopathy	Not reported	1,580	Chemotherapy	Clinical improvement	Not reported
González-Paredes et al.	1	12/F	Nodular lymphocyte-predominant HL	Abdominal mass, renal involvement	Not reported	27,552	Chemotherapy	Partial response	Renal impairment at diagnosis
Cyriac et al.	1	8/M	Mixed cellularity HL	Mediastinal disease, SVC syndrome	Advanced	14,220	ABVD chemotherapy	Good response	Favorable outcome
Voeller et al.	2	Pediatric	Not HL (hematologic malignancies)	Systemic involvement	Not applicable	Severe hypereosinophilia	Targeted therapy/chemotherapy	Variable	Complications reported
Present case	1	17/F	NS-cHL subtype	Cervical, mediastinal, abdominal, and splenic involvement	IIIBs	97,880	OEPA/COPDAC + radiotherapy	Partial metabolic response (Deauville 4)	Complete metabolic response; alive, under surveillance

Although eosinophilia has been described in Hodgkin lymphoma, cases with such extreme levels are rarely reported. The patient presented with anxiety, pruritus, palmoplantar rash, diaphoresis, splenomegaly, and marked lymphadenopathy, accompanied by an extraordinarily high eosinophil count. This unusual presentation underscores the value of considering HL in the differential diagnosis of unexplained hypereosinophilia, particularly in pediatric and adolescent patients. As a limitation, complete retrieval of all imaging reports was not possible due to the retrospective nature of this case. However, the consistency between imaging interpretation, clinical evolution, and hematologic response—particularly the sustained normalization of eosinophil counts—supports the reliability of the reported complete metabolic response.

This case adds to the scarce literature on profound hypereosinophilia occurring in association with HL in pediatric patients. It underscores the importance of considering malignant etiologies in the differential diagnosis of unexplained eosinophilia and highlights the value of prompt, comprehensive evaluation. Further research is needed to clarify the underlying mechanisms and potential prognostic implications of this rare association.

## Data Availability

The original contributions presented in the study are included in the article/Supplementary Material, further inquiries can be directed to the corresponding author.
